# Examining gender differences in gait parameters between non-smoker and smoker participants

**DOI:** 10.25122/jml-2022-0347

**Published:** 2023-05

**Authors:** Mohammad Ahsan, Turki Abualait, Shibili Nuhmani, Mohammed Al-Subaiei, Maitha Aldokhayyil

**Affiliations:** 1. Department of Physical Therapy, College of Applied Medical Sciences, Imam Abdulrahman Bin Faisal University, Dammam, Kingdom of Saudi Arabia

**Keywords:** gait parameters, cadence, stride length, stride velocity, gait cycle, stance phase

## Abstract

Smoking is one of the predictors of decreased cardiopulmonary endurance. Gait disturbance may be due to many reasons, including cardiovascular endurance. This study aimed to determine differences in gait parameters between non-smoker and smoker participants. A cross-sectional design was employed, involving thirty non-smokers and thirty-seven smokers as participants. Detailed interviews were conducted to gather information on smoking habits, status, and history. Gait parameters were measured using a high-quality 3D accelerometer, 3D gyroscope, and barometric pressure sensors (Physilog4 from GaitUp). Anthropometric characteristics were described, and mean values with standard deviations (SD) were calculated. An independent two-tailed t-test was conducted to compare gait parameters between non-smokers and smokers, with statistical significance set at p<0.05. The analysis revealed significant differences in various gait parameters between non-smokers and smokers. Specifically, significant differences were found in cadence (t=9.95, p=0.001), stride length (t=6.85, p=0.001), stride velocity (t=-6.58, p=0.001), stance (t=2.02, p=0.001), swing (t=3.46, p=0.001), foot flat (t=-8.94, p=0.001), pushing (t=3.53, p=0.001), and double support (t=-13.35, p=0.001). However, no significant difference was found between non-smokers and smokers in the loading phase (t=-1.57, p= 0.121). There were significant differences in general and temporal gait parameters between smokers and non-smokers. Gait parameters provide valuable insights for evaluating functional performance and providing objective and quantitative data to assess gait disorders. Future studies should include longitudinal studies with large sample sizes to explore the effects of potential confounders on gait parameters.

## INTRODUCTION

Smoking is one of the leading causes of mortality and morbidity. According to the Saudi Ministry of Health, smoking kills 71 men and 21 women each week, equating to 5,000 deaths per year due to smoking-related ailments. Smoking has been linked to various serious health conditions, including cancer, heart disease, stroke, chronic obstructive pulmonary disease (COPD), diabetes, and even erectile dysfunction in males [1]. In accordance with the World Health Organization framework convention, the Saudi Ministry of Health addressed the state of tobacco usage in Saudi Arabia in a national tobacco control program, indicating that the average age of initiation was 19 years. They also confirmed that the possibility of being a smoker increases with age [2]. Studies revealed that smoking causes lung cancer, chronic respiratory and cardiovascular diseases, a risk factor for respiratory infections, worse surgical outcomes, osteoporosis, delayed wound healing, diabetes, reproductive abnormalities, and duodenal and gastric ulcers [3,4]. Vital organs, such as the heart, brain, and neurological tissues, are negatively affected by smoking. Smoking harms an individual's physical, mental, and social qualities and general health [5,6].

The most prevalent human movement is gait, a voluntary and cyclical form of vertical mobility characterized by the alternating and progressive action of the lower limbs [7]. Gait is a high-level, attention-demanding, and precisely controlled task [8]. Normal gait requires sensory integration (vestibular, proprioceptive, and visual), motor planning and execution (planning, initiation, automatization, integration, and coordination of gait), and integrated musculoskeletal and cardiovascular systems [9]. In gait analysis, general parameters such as cadence, stride length, stride velocity, and gait cycle are examined to assess walking patterns and identify potential causes of gait disorders. By analyzing gait, specific abnormalities can be identified, providing valuable insights into their underlying causes [10]. Gait abnormalities may be caused by impaired muscle weakness, irregular joint position, static or dynamic muscle contracture, abnormal muscular tone, and cardiovascular condition [11].

Cigarette smoking is a well-known and significant risk factor for various cardiovascular diseases, encompassing a range of disorders affecting the heart and blood vessels [12]. Smoking exposes the body to chemicals in smoke-contaminated oxygen, leading to detrimental effects on the cardiovascular system [13]. Reduced cardiopulmonary endurance is among the predictors associated with smoking, demonstrated by lower peak oxygen intake levels during treadmill exercise testing [5,6]. When combined with physiological changes, adverse health conditions can disrupt normal gait patterns [14]. Smoking has always been associated with a reduced walking distance during the well-established 6-minute walk test [15]. Furthermore, research has shown that increased pack years of smoking are associated with poorer global gait parameters, characterized by slower and smaller steps, longer double support, and altered rhythm and pace in the community-dwelling population [16]. Gait is considered an accurate reflection of general health [7].

As a multidimensional phenomenon, gait can be thoroughly studied using various techniques. Gait analysis encompasses the exploration of multiple interacting characteristics with the aim of better understanding potential disruptions or limitations. The objective of this study was to compare the general and temporal gait parameters between non-smokers and smokers. The relationship between smoking and particular spatiotemporal metrics may indicate the processes underlying their relationships with gait. This knowledge can lead to the identification of improved intervention options to prevent future gait disturbances in smokers and non-smokers. Such information may be valuable when considering rehabilitation strategies or making complex therapeutic decisions.

## MATERIAL AND METHODS

### Study design and location

This cross-sectional study was conducted between May 2022 and October 2022 at the Department of Physical Therapy, Imam Abdulrahman Bin Faisal University, located in Dammam, Kingdom of Saudi Arabia.

### Sample size calculation

The sample size was calculated using G*Power (3.1.9.4) software. The parameters used for calculation were as follows: test family - t-test, statistical test - the difference between two independent means (two groups), a priori power analysis - tails: two, effect size: 0.8, α error probability: 0.05, power (1-β error probability): 0.85. The calculated sample size was 60 (30 participants in each group). To account for potential dropouts and reduce sampling error, the sample size was increased by 10%.

### Screening

Detailed interviews were conducted to screen potential participants. Individuals with a history of drug use, musculoskeletal ailments, cardiovascular diseases, or neurological problems that could impact normal gait patterns were excluded from the study.

### Participants

A convenience sample of 67 participants, comprising 30 non-smokers and 37 smokers, was recruited for the study. The sample included 41 males and 26 females. Participants' age ranged from 22 to 32 years, height ranged from 162 to 181 cm, weight ranged from 65 to 95 kg, and body mass index ranged from 22 to 25 kg/m². Non-smokers had never smoked, while current smokers had a smoking history of two or more years.

### Experimental tools

A portable electronic self-calibrated stadiometer cum weighing scale (Detecto scale, 750 USA) was used to the measured height, weight, and body mass index of each participant. Physilog 4 from GaitUp (SA., Lausanne, Switzerland) was utilized to determine participants' gait parameters. The Physilog from GaitUp is a valid and reliable instrument for measuring spatiotemporal gait characteristics [17,18].

### Experimental setup

The high-quality 3D accelerometer, 3D gyroscope, and barometric pressure sensor (Physilog4 from GaitUp) were securely attached to the participants' upper feet using straps to ensure stability during measurements. It provided raw 3D acceleration data at a sample rate of 128 Hz. To measure synchronized Physilog data, the left and right leg sensors were turned on and connected to the network using the main button within a short time interval. Each sensor began recording data upon pressing the start button and synchronized with the master sensor. The green light on each sensor started blinking to indicate synchronization with the master sensor. The measurement was initiated by pressing the main button on the sensors and stopped by pressing the button until the LED turned orange.

### Gait recording

The gait recording procedure was standardized for each participant in the study. The examination technique began with an interview of each participant, followed by the completion of self-administered questionnaires at their own convenience. A 20-meter straight, flat, and hard surface was chosen for the walk. Physilog inertial sensors were attached to the foot of each participant. Participants were instructed to walk at their own pace on the straight path. The Physilog application for gait analysis was installed on a tablet used by the researcher, and a Bluetooth connection was established with the sensors. The previously described experimental setup was utilized to record three trials for each participant. The average of three trials was calculated and used for further analysis. The variables of interest in this study included general parameters of gait (cadence in steps/min, stride length in meters, stride velocity in meters/second) and temporal parameters of gait (stance phase as a percentage of the gait cycle, swing phase as a percentage of the gait cycle, loading phase as a percentage of stance, foot flat as a percentage of stance, pushing as a percentage of stance, and double support as a percentage of the gait cycle).

### Statistical analysis

The data analysis was performed using the Statistical Package for the Social Sciences (SPSS), version 26.0, developed by SPSS Inc., located in Chicago, IL, United States. Data were carefully examined before the analysis to identify outliers and missing values. The normality of the data distribution was assessed using the Shapiro-Wilk test, with a significance level at p>0.05. The anthropometric characteristics and gait parameters were analyzed using the independent t-test (two-tails). A p-value of ≤0.05 was considered statistically significant.

## RESULTS

### Anthropometric characteristics

Table 1 shows the anthropometric characteristics of non-smoker and smoker participants. The mean age of non-smokers was 26.03±5.54 years, whereas smokers had a mean age of 24.76±4.40 years. Non-smokers had a mean height of 178.03±4.89 cm, and smokers had a mean height of 175.21±5.94 cm. The mean weight for non-smokers was 74.9±10.08 kg, while smokers had a mean weight of 70.46±8.58 kg. In terms of body mass index (BMI), non-smokers had a mean BMI of 23.62±2.59 kg/m², and smokers had a mean BMI of 23.18±2.07 kg/m². There were no significant differences in anthropometric characteristics between non-smokers and smokers, indicating similar profiles in terms of age, height, weight, and BMI. Table 2 also revealed that there were no significant differences between male and female participants in terms of anthropometric characteristics.

**Table 1.** Anthropometric characteristics of non-smoker and smoker participants

		N	Mean±Std. Deviation	Std. Error Mean	t	Sig. (2-tailed)
Age (Years)	Non-smokers	30	26.03±5.54	.82	-.660	.512
	Smokers	37	24.76±4.40	.72		
Height (cm)	Non-smokers	30	178.03±4.89	.89	1.944	.054
	Smokers	37	175.21±5.94	1.41		
Weight (Kg)	Non-smokers	30	74.9±10.08	1.84	1.959	.052
	Smokers	37	70.46±8.58	1.14		
BMI (kg/m^2^)	Non-smokers	30	23.62±2.59	.47	.740	.462
	Smokers	37	23.18±2.07	.34		

**Table 2.** Anthropometric characteristics of male and female participants

	Gender	N	Mean±Std. Deviation	Std. Error Mean	t	Sig. (2-tailed)
Age	Male	41	27.00±4.604	.667	1.81	.913
	Female	26	25.00±4.272	.903		
Height	Male	41	179.54±6.201	.968	5.13	.623
	Female	26	173.19±4.924	.966		
Weight	Male	41	75.42±10.182	1.590	6.53	0.88
	Female	26	69.24±7.741	1.518		
BMI	Male	41	24.04±8.064	1.259	.541	.065
	Female	26	22.97±6.172	1.211		

### Genders-specific analysis

When comparing genders separately, there were significant differences in cadence (p=0.001), stride length (p=0.001), stride velocity (p=0.001), swing (p=0.001), flat foot (p=0.001), pushing (p=0.030), and double support (p=0.001) between non-smoker males and smoker males (Table 3). However, male participants had no significant differences in the stance phase (p=0.656) and loading phase (p=0.552). Non-smoker males had higher cadence, longer stride length, longer stance, larger swing phase, and greater pushing compared to male smokers.

**Table 3.** Comparison of gait parameters between non-smoker and smoker male participants

Parameters	Male	N	Mean±Std. Deviation	Std. Error Mean	t	Sig. (2-tailed)
Cadence (Steps/Min)	Non-Smokers	18	113.28±5.07	1.20	7.47	0.001*
	Smokers	23	103.64±3.15	0.66		
Stride lenght (Meters)	Non-Smokers	18	1.33±0.07	0.02	5.20	0.001*
	Smokers	23	1.24±0.05	0.01		
Stride velocity (Meters/Sec)	Non-Smokers	18	1.06±0.07	0.02	-5.62	0.001*
	Smokers	23	1.18±0.07	0.02		
Stance (%Cycle)	Non-Smokers	18	61.30±2.15	0.51	0.45	0.656
	Smokers	23	60.99±2.17	0.45		
Swing (%Cycle)	Non-Smokers	18	40.18±1.47	0.35	3.64	0.001*
	Smokers	23	38.08±2.07	0.43		
Loading (%Stance)	Non-Smokers	18	14.69±4.72	1.11	-0.61	0.552
	Smokers	23	15.69±5.71	1.19		
Foot flat (%Stance)	Non-Smokers	18	37.14±7.53	1.77	-6.83	0.001*
	Smokers	23	55.10±9.32	1.94		
Pushing (%Stance)	Non-Smokers	18	42.47±9.94	2.34	2.25	0.030*
	Smokers	23	35.62±9.44	1.97		
Double support (%Cycle)	Non-Smokers	18	17.97±2.34	0.55	-10.08	0.001*
	Smokers	23	23.96±1.45	0.30		

*Significant at ≤0.05 level.

In the female subgroup, significant differences were observed in cadence (p=0.001), stride length (p=0.001), stride velocity (p=0.001), stance (p=0.005), foot flat (p=0.001), pushing (p=0.008), and double support (p=0.001) (Table 4). In contrast, female non-smokers and smokers had no significant difference in the swing (p=0.247) and loading phase (p=0.075). Additionally, non-smoker females had higher cadence, stride length, stance, swing, and pushing phase values than smoker females. Smoker females had higher stride velocity, loading, flat foot, and double support scores than non-smoker females.

**Table 4.** Comparison of gait parameters between non-smoker and smoker female participants

Parameters	Female	N	Mean±Std. Deviation	Std. Error Mean	t	Sig. (2-tailed)
Cadence (Steps/Min)	Non-Smokers	12	112.16±3.81	1.10	6.59	0.001*
	Smokers	14	102.59±3.59	0.96		
Stride lenght (Meters)	Non-Smokers	12	1.32±0.05	0.01	4.56	0.001*
	Smokers	14	1.22±0.06	0.02		
Stride velocity (Meters/Sec)	Non-Smokers	12	1.10±0.07	0.02	-3.91	0.001*
	Smokers	14	1.23±0.10	0.03		
Stance (%Cycle)	Non-Smokers	12	62.35±1.37	0.40	2.99	0.005*
	Smokers	14	60.23±2.10	0.56		
Swing (%Cycle)	Non-Smokers	12	39.12±2.73	0.79	1.19	0.247
	Smokers	14	38.14±1.38	0.37		
Loading (%Stance)	Non-Smokers	12	12.65±2.84	0.82	-1.86	0.075
	Smokers	14	16.00±5.62	1.50		
Foot flat (%Stance)	Non-Smokers	12	37.77±7.74	2.23	-5.86	0.001*
	Smokers	14	55.86±7.95	2.12		
Pushing (%Stance)	Non-Smokers	12	42.29±12.01	3.47	2.87	0.008*
	Smokers	14	31.68±6.38	1.71		
Double support (%Cycle)	Non-Smokers	12	18.33±1.85	0.53	-8.65	0.001*
	Smokers	14	24.46±1.76	0.47		

*Significant at ≤0.05 level.

### Comparison between genders

When comparing genders, significant differences were observed across all gait parameters (cadence, stride length, stride velocity, stance, loading, foot flat, pushing, and double support), except for the swing phase (Table 5). Male participants had higher mean values of cadence, stride length, stance, swing, and pushing than females. Female participants had greater mean values of loading, foot flat, and double support than male participants.

**Table 5.** Comparison of gait parameters between male and female participants

Parameters	Gender	N	Mean±Std. Deviation	Std. Error Mean	t	Sig. (2-tailed)
Cadence (Steps/Min)	Male	41	111.36±4.72	0.75	9.38	0.001*
	Female	26	101.87±2.80	0.54		
Stride lenght (Meters)	Male	41	1.31±0.06	0.01	5.76	0.001*
	Female	26	1.22±0.05	0.01		
Stride velocity (Meters/Sec)	Male	41	1.09±0.07	0.01	-7.52	0.001*
	Female	26	1.22±0.08	0.02		
Stance (%Cycle)	Male	41	61.83±2.15	0.34	3.43	0.005*
	Female	26	60.16±1.60	0.31		
Swing (%Cycle)	Male	41	39.11±2.18	0.34	1.26	0.213
	Female	26	38.45±1.95	0.38		
Loading (%Stance)	Male	41	13.53±4.38	0.69	-2.93	0.005*
	Female	26	17.03±5.34	1.03		
Foot flat (%Stance)	Male	41	40.85±10.24	1.62	-6.98	0.001*
	Female	26	56.93±7.54	1.45		
Pushing (%Stance)	Male	41	41.76±10.29	1.63	4.26	0.001*
	Female	26	32.01±7.24	1.39		
Double support (%Cycle)	Male	41	19.12±2.57	0.41	-10.98	0.001*
	Female	26	24.89±1.08	0.21		

*Significant at ≤0.05 level.

### Gait parameters

Table 6 revealed significant differences between non-smokers and smokers in various gait parameters. Significant differences were found for cadence (t=9.95, p=0.001), stride length (t=6.85, p=0.001), stride velocity (t=-6.58, p=0.001), stance (t=2.02, p=0.001), swing (t=3.46, p=0.001), foot flat (t=-8.94, p=0.001), pushing (t=3.53, p=0.001), and double support (t=-13.35, p=0.000). However, no significant difference was observed for the loading phase (t=-1.57, p=0.121). Non-smokers exhibited higher cadence, longer stride length, longer stance, larger swing phase, and greater pushing compared to smokers. Conversely, smokers had higher stride velocity, greater loading phase, larger foot flat, and increased double support compared to non-smokers.

**Table 6.** Comparison of gait parameters between non-smoker and smoker participants

Parameters	Gender	N	Mean±Std. Deviation	Std. Error Mean	t	Sig. (2-tailed)
Cadence (Steps/Min)	Non-Smokers	30	112.83±4.57	0.83	9.95	0.000*
	Smokers	37	103.24±3.31	0.54		
Stride lenght (Meters)	Non-Smokers	30	1.32±0.06	0.01	6.85	0.000*
	Smokers	37	1.23±0.05	0.01		
Stride velocity (Meters/Sec)	Non-Smokers	30	1.07±0.07	0.01	-6.58	0.000*
	Smokers	37	1.20±0.08	0.01		
Stance (%Cycle)	Non-Smokers	30	61.72±1.92	0.35	2.02	0.048*
	Smokers	37	60.70±2.15	0.35		
Swing (%Cycle)	Non-Smokers	30	39.76±2.09	0.38	3.46	0.001*
	Smokers	37	38.10±1.82	0.30		
Loading (%Stance)	Non-Smokers	30	13.88±4.14	0.76	-1.57	0.121
	Smokers	37	15.81±5.60	0.92		
Foot flat (%Stance)	Non-Smokers	30	37.39±7.49	1.37	-8.94	0.001*
	Smokers	37	55.39±8.72	1.43		
Pushing (%Stance)	Non-Smokers	30	42.40±10.61	1.94	3.53	0.001*
	Smokers	37	34.13±8.54	1.40		
Double support (%Cycle)	Non-Smokers	30	18.11±2.13	0.39	-13.35	0.000*
	Smokers	37	24.15±1.57	0.26		

*Significant at ≤0.05 level.

## DISCUSSION

The study aimed to determine differences in gait parameters between non-smokers and smokers. The results revealed significant differences between these two groups in gait parameters. Non-smokers had a higher cadence, longer stride length, longer stance, larger swing phase, and larger pushing than smokers. Smokers had higher stride velocity, higher loading phase, greater foot flat, and bigger double support than non-smokers.

Furthermore, when comparing genders, significant differences were observed in all gait parameters (cadence, stride length, stride velocity, stance, loading, foot flat, pushing, and double support) except for the swing phase. Male participants had greater mean values of cadence, stride length, stance, swing, and pushing than females. Female participants had greater mean values of loading, foot flat, and double support than male participants.

These findings are consistent with previous studies investigating gender-specific differences in gait parameters. Their findings are aligned with our findings as young, healthy females have shorter stride lengths and slower gait speed while walking at their own pace than healthy young males, largely due to their shorter height [17]. A study revealed that the spatiotemporal parameters of normal gait speed, gait cycle, stride length, and cadence did not differ significantly between males and females [18]. Males had higher stride length, step time, cadence, and walking speed than females [19]. Another study found that while females have longer normalized stride lengths and higher cadence, both genders have the same step width and walking velocity, suggesting that females make efforts to expand their stride length to match the pace of males [20]. Moreno *et al*. proposed that race may influence gender differences in gait characteristics, even though there were no significant differences in spatiotemporal parameters between genders [21]. Smith *et al*. revealed no differences in spatiotemporal parameters between females and males. Step and stride lengths were statistically higher in females than in males [22]. We believe all these differences were due to the physical structure differences between genders.

Our study findings are consistent with the research conducted by Verlinder *et al*. [16], who investigated the relationship between tobacco consumption and gait. They found that smoking habits were associated with worse gait velocity and global gait. Similarly, our findings indicated that smokers had poorer stride length, stride velocity, duration of the gait cycle, swing phase, loading phase, and pushing compared to non-smokers. In contrast, non-smokers demonstrated higher values in the stance phase, foot flat, and double support. Different pathways and mechanisms may describe the possible differences between smokers and non-smokers with gait. It is evident in epidemiological and animal studies that smoking and its components like nicotine, tar, carbon dioxide, and carbon monoxide affect various organs, including the nervous, cardiovascular, and musculoskeletal systems [23–25]. Better functioning of these organs and systems could improve gait parameters [25–27].

One investigation found that greater pack-years of smoking were associated with poorer gait phases and pace, resulting in slower and smaller steps with longer double support [16]. Similarly, our findings support these observations as smokers exhibited longer stance phase, foot flat, and double support durations compared to non-smokers. These differences indicate that smokers had slower gait velocity, as it took them more time to complete each gait cycle.

A study found significant gait speed impairment among women associated with smoking status [28]. Current smokers have lower peak oxygen uptake than individuals who have never smoked, with decreases of 15% and 7% reported [29,30]. Smokers had a lower assessment of their capacity to walk various distances and speeds and climb stairs than non-smokers, which may contribute to their lower daily physical activity in the community [31].

Following a physical exercise intervention for smokers and non-smokers, improvements in the walking economy were observed, with a 9% increase in smokers and an 11% increase in non-smokers. Significant differences were found between smokers and non-smokers for the total walking distance during the six-minute walk test, pain-free walking distance, and daily physical activities [32]. Additionally, smoking is associated with poor physical performance in older women, as indicated by slower walk tests and difficulties with rising from a seated position [33].

Smoking impacts physical performance by affecting vascular function and metabolism [34]. In addition, it reduces muscle protein synthesis and raises the expression of genes linked to impaired muscle maintenance [35]. It also reduces oxygen flow to muscle groups, which can immediately impact physical performance [36]. The acute effect includes minimum endurance, lower maximum heart rates, and muscle fatigue [37,38]. Given the magnitude of these negative effects, smoking may obscure the association between general and temporal gait parameters in both male and female participants. In addition, there were significant differences in gait parameters between smokers and non-smokers. This suggests that non-smoking is vital to maintaining proper physical function. Smokers may remain at high risk, and smoking habits decelerate physical function due to the harmful effect of smoking. The findings of this study demonstrate differences in gait parameters between male and female smokers and non-smokers. Health policymakers must take proactive measures to promote better health and enhance the overall quality of life. These measures should focus on encouraging individuals to engage in daily physical activities and raising awareness about the dangers of smoking.

This study had some limitations. First, its cross-sectional design precludes establishing a causal relationship between anticipated factors and functional performance outcomes. Second, the sample size was smaller than in other epidemiological and prevalence studies, which may account for the statistically insignificant results. The authors did not examine smoking history, which may have affected the outcome variable favorably. Despite these limitations, the study has notable strengths. The gait characteristics were measured using valid and reliable tools, ensuring the accuracy of the data collected. In clinical settings, these gait parameters can be considered vital signs.

## CONCLUSION

Our results showed significant differences in the overall and temporal gait parameters between non-smokers and smokers. Non-smokers had a higher cadence, longer stride length, longer stance, larger swing phase, and larger pushing than smokers. Smokers had higher stride velocity, higher loading phase, greater foot flat, and bigger double support than non-smokers. Functional performance evaluations and quantitative data on gait disorders benefit greatly from gait characteristics. Longitudinal studies with high sample numbers are needed in future research to investigate the impact of various confounders on gait parameters.
